# Hypothalamic orexinergic neuron changes during the hibernation of the Syrian hamster

**DOI:** 10.3389/fnana.2022.993421

**Published:** 2022-09-09

**Authors:** Jesús M. López, Paula Carballeira, Javier Pozo, Gonzalo León-Espinosa, Alberto Muñoz

**Affiliations:** ^1^Departamento de Biología Celular, Universidad Complutense, Madrid, Spain; ^2^Departamento de Química y Bioquímica, Facultad de Farmacia, Universidad San Pablo-Centro de Estudios Universitarios (CEU), Madrid, Spain; ^3^Laboratorio Cajal de Circuitos Corticales, Centro de Tecnología Biomédica (CTB), Universidad Politécnica de Madrid, Madrid, Spain; ^4^Instituto Cajal, Consejo Superior de Investigaciones Científicas (CSIC), Madrid, Spain

**Keywords:** Golgi apparatus, Golgi fragmentation, orexins, hypothalamus, GM-130, torpor, C-Fos, hypocretins

## Abstract

Hibernation in small mammals is a highly regulated process with periods of torpor involving drops in body temperature and metabolic rate, as well as a general decrease in neural activity, all of which proceed alongside complex brain adaptive changes that appear to protect the brain from extreme hypoxia and low temperatures. All these changes are rapidly reversed, with no apparent brain damage occurring, during the short periods of arousal, interspersed during torpor—characterized by transitory and partial rewarming and activity, including sleep activation, and feeding in some species. The orexins are neuropeptides synthesized in hypothalamic neurons that project to multiple brain regions and are known to participate in the regulation of a variety of processes including feeding behavior, the sleep-wake cycle, and autonomic functions such as brown adipose tissue thermogenesis. Using multiple immunohistochemical techniques and quantitative analysis, we have characterized the orexinergic system in the brain of the Syrian hamster—a facultative hibernator. Our results revealed that orexinergic neurons in this species consisted of a neuronal population restricted to the lateral hypothalamic area, whereas orexinergic fibers distribute throughout the rostrocaudal extent of the brain, particularly innervating catecholaminergic and serotonergic neuronal populations. We characterized the changes of orexinergic cells in the different phases of hibernation based on the intensity of immunostaining for the neuronal activity marker C-Fos and orexin A (OXA). During torpor, we found an increase in C-Fos immunostaining intensity in orexinergic neurons, accompanied by a decrease in OXA immunostaining. These changes were accompanied by a volume reduction and a fragmentation of the Golgi apparatus (GA) as well as a decrease in the colocalization of OXA and the GA marker GM-130. Importantly, during arousal, C-Fos and OXA expression in orexinergic neurons was highest and the structural appearance and the volume of the GA along with the colocalization of OXA/GM-130 reverted to euthermic levels. We discuss the involvement of orexinergic cells in the regulation of mammalian hibernation and, in particular, the possibility that the high activation of orexinergic cells during the arousal stage guides the rewarming as well as the feeding and sleep behaviors characteristic of this phase.

## Introduction

Hibernation in small mammals, such as the Syrian hamster, is a state that has evolved to allow the animal to survive periods of adverse environmental conditions with low energy availability (Humphries et al., 2003) and is characterized by periods of torpor with reduced metabolic rate and body temperature that can last several days, interspersed with short arousal periods of activity and normothermia (Frerichs et al., 1994; Geiser, 2004, 2013; Drew et al., 2007; Geiser and Martin, 2013). Hibernation requires drastic physiological changes to maintain energy savings (Geiser, 2004, 2013; Geiser and Martin, 2013) and results in a decrease in metabolic rate (Zhou et al., 2001). During torpor bouts, energetically demanding cellular processes like transcription and protein synthesis are severely reduced, with such processes recovering fully during the interbout arousal (Zhegunov, 1988; Rolfe and Brown, 1997; van Breukelen and Martin, 2001; Osborne et al., 2004). However, whether or not global inhibition of transcription occurs remains unclear (Carey and Martin, 1996; Hittel and Storey, 2002) and transcription modulation during torpor seems to be tissue dependent (Soukri et al., 1996; van Breukelen and Martin, 2001). Torpor state during hibernation is characterized by a virtual cessation of neuronal activity in the cortical and midbrain areas (Strumwasser, 1959; South, 1972; Walker et al., 1977; Igelmund, 1995). Although the neural circuits involved in the regulation of mammalian hibernation have not been fully characterized, the onset of the torpor phase of hibernation is thought to involve hypothalamic activation and inhibition of the cerebral cortex, whereas arousals are induced by hypothalamic functions (Mihailovic et al., 1968; Kilduff et al., 1989, 1993; Bitting et al., 1994; O’Hara et al., 1999; Ruby et al., 2002).

Orexins A and B (also known as hypocretins 1 and 2, respectively) are two neuropeptides with 33 and 28 amino acids, respectively, and are synthesized in cells of the hypothalamus that were initially found in the human brain as modulators of feeding behavior (de Lecea et al., 1998; Sakurai et al., 1998). Orexins are synthesized as prepro-orexin (with 130 amino acids in rodents) in the rough endoplasmic reticulum of orexinergic cells and are subsequently processed in the Golgi complex. After being released into the synaptic gap, orexins act on the postsynaptic neurons but can also diffuse to act at long distances (of the order of micrometers) on adjacent neurons and astrocytes, since there are no reuptake systems for neuropeptides, and they remain in the extracellular medium until they are degraded by peptidases (van den Pol, 2012). Therefore, the action of orexins generally involves lateral synapses that modulate the connections between neurons of other neurotransmitter systems like cholinergic and monoaminergic systems (Leonard and Kukkonen, 2014). Orexins activate two G protein-coupled receptors (OX1R, selective for orexin A, and OX2R with a similar affinity for both orexins) located pre- and post-synaptically, with excitatory effects following an increase in the intracellular Ca2+ concentration (Johansson et al., 2008; Kukkonen and Leonard, 2014).

The orexinergic system is involved in the regulation of behavioral states (Sakurai, 2007; Zhang et al., 2010; Sellayah and Sikder, 2013; Kuwaki, 2015). The system integrates metabolic, circadian and limbic inputs, conveying this information to a network of monoamines and neuromodulators (de Lecea and Huerta, 2014). In mammals, orexinergic cells are generally located in a discrete position in the lateral hypothalamus, but they receive and integrate inputs from a wide variety of brain regions including limbic system, hypothalamus, thalamus, brainstem cholinergic nuclei, reticular formation, and midbrain raphe nuclei (de Lecea, 2015). These cells sense more than 10 neurotransmitters and hormones [see Hara et al. (2009) and Inutsuka and Yamanaka (2013)] through a wide variety of receptors (Sakurai, 2007). In addition, orexinergic cells give rise to an extensive network of orexinergic fibers throughout all major brain subdivisions, representing the efferent projections of these hypothalamic neurons (Broberger et al., 1998; de Lecea et al., 1998; Sakurai et al., 1998; Dube et al., 1999; Nambu et al., 1999; Mintz et al., 2001; Zhang et al., 2001; Nixon and Smale, 2007; Kruger et al., 2010; Bhagwandin et al., 2011; Gravett et al., 2011; Dell et al., 2013). Thus, orexin innervation has been mainly described in the cerebral cortex, nucleus accumbens, septal area, amygdala, preoptic area, paraventricular and arcuate hypothalamic nuclei, thalamus, solitary tract nucleus, and especially in monoaminergic and cholinergic centers that are involved in the sleep-wake cycle and reward system, such as the tuberomamillary hypothalamic nucleus, ventral tegmental area, substantia nigra, pedunculopontine and laterodorsal tegmental nuclei, locus coeruleus, and the raphe nuclei (Peyron et al., 1998; Cutler et al., 1999; Date et al., 1999; Shibata et al., 2008; Peyron and Kilduff, 2017; Wang et al., 2018). Double immunolabeling studies have indicate that both orexins colocalize in the same hypothalamic neurons and in their fiber projections (Zhang et al., 2004).

Studies carried out in non-hibernating mammalian species have demonstrated the involvement of orexins in the control of adenohypophyseal hormone release; autonomic functions including stimulation of gastrointestinal functions, heart rate and arterial pressure regulation; and energy homeostasis including feeding behavior, maintenance of body temperature and energy expenditure on brown adipose tissue non-shivering thermogenesis—the main sympathetic nervous system-mediated mechanism to regain body temperature after hypothermia. In addition, orexins have been shown to be involved in the induction and maintenance of arousal and the control of the sleep-wakefulness cycle, with implications in pathologies such as narcolepsy (Mieda and Sakurai, 2012; Zhang et al., 2013; Li and de Lecea, 2020; Toor et al., 2021). Many of these important functions are particularly regulated during the different phases of the hibernation cycle and although previous studies have suggested the involvement of the orexinergic system in this regulation (Tamura et al., 2011; Schwartz et al., 2013), the orexinergic system has not been studied in detail in mammalian hibernating species. In the present study, we hypothesized that orexinergic neurons might be involved in the regulation of hibernation and that their activation might vary across the hibernation cycle. Here, we analyzed the distribution of orexinergic neurons and fibers in the brain of the Syrian hamster, in particular their relationship with catecholaminergic and serotonergic centers. In addition, we indirectly characterized the changes in activity of orexinergic cells in the different phases of hibernation by analyzing the intensity of immunolabeling of orexin A and the neuronal activity marker C-Fos. Finally, the size of the Golgi apparatus (GA) has been related to the level of cell activity (Lucassen et al., 1993; Salehi et al., 1994) and during hibernation, the GA of different cell types—including hippocampal and neocortical neurons (Popov et al., 1999; Bocharova et al., 2011; Antón-Fernández et al., 2015; León-Espinosa et al., 2019)—undergo a pronounced morphological reorganization including fragmentation and volume decrease during torpor, with a rapid rebuilding during arousals. Therefore, in the present study, we characterized the integrity of the GA of orexinergic neurons throughout hibernation, as revealed by the expression of the GA marker GM-130, a structural GA protein that participates in the protein complex that promotes the stacking and lateral tethering of Golgi cisternae for ribbon formation (Nakamura et al., 1995; Barr et al., 1997, 1998; Shorter and Warren, 1999; Puthenveedu and Linstedt, 2001, 2005; Puthenveedu et al., 2006; Nakamura, 2010).

The present results revealed that, during the hibernation phases, orexinergic cells undergo selective structural and neurochemical changes which are likely to be related to the regulation of torpor state, and to rewarming, as well as the feeding and sleep behaviors, all of which are characteristic of arousals from torpor.

## Materials and methods

In the present study, we used 25 male 3-month-old Syrian hamsters (*Mesocricetus auratus*) that were purchased from Janvier Labs (Le Genest-Saint-Isle, France). All experimental procedures were carried out at the animal facility of the Cajal Institute in Madrid (registration number ES 28079 0000184) and were approved by the institutional Animal Experiment Ethics Committee (PROEX 292/15). The animals had free access to food and water and were kept at 23°C with an 8:16-h light/dark cycle in our animal facility until induction of hibernation (torpor and arousal experimental groups) or until their sacrifice (control animals). After the acclimatization period (4–6 weeks), 15 out of the 25 animals were transferred to a chamber to artificially induce hibernation. The chamber controls the temperature and illumination, while also allowing the hamsters to be monitored by measuring their locomotor activity. The animals spent a week in this chamber where light was progressively dimmed (until complete darkness) and the temperature decreased (from 23 to 4°C). The torpor stage was considered to have started when the animals showed periods of inactivity of 24 h, which generally occurred 2–3 months after the chamber temperature had reached 4°C. We considered animals to be torpid only when they had completed three full bouts of torpor (3–4 days of inactivity) before they were sacrificed. Since a torpor bout lasts for 3–4 days, we sacrificed the animals on the second day after torpor started—to ensure that the animals were in deep torpor. The arousal experimental group was obtained by removing the torpid animals from the hibernation chamber and inducing their awakening 1–1.5 h before being sacrificed. The temperature of each animal was checked using an infrared thermometer before perfusion to ensure that they were in deep torpor (*n* = 7, temperature: 5–7.5°C) or in arousal (*n* = 9, temperature: 32–35°C). Control animals (*n* = 9, temperature: 36.5–37°C) were not transferred to the hibernation chamber [see Antón-Fernández et al. (2015) for further details]. All animals were sacrificed by a lethal intraperitoneal injection of sodium pentobarbital (200 mg/kg) and then perfused intracardially with saline solution followed by 4% paraformaldehyde in phosphate buffer (PB; 0.1 M; pH 7.4).

### Bright field immunolocalization of orexins

The brains of two animals from the control group were used to study immunohistochemically the distribution of orexinergic neurons within the central nervous system of *M. auratus*. The brain of each animal was removed and post-fixed by immersion in the same fixative for 2–3 h at 4°C. After rinsing in PB, the brains were cryoprotected in 30% sucrose solution in 0.1M PB for 10 h and later embedded in a block of 20% gelatin and 30% sucrose solution that was subsequently fixed in 10% formaldehyde in PB for 6 h at 4°C. The blocks were cut with a freezing sliding microtome (Microm, Walldorf, Germany). Serial sections (40 μm thick) were stored in ethyleneglycol/glycerol at -20°C until they were used. Free-floating sections were pretreated with 1% H_2_O_2_ in PB for 30 min to eliminate the endogenous peroxidase activity and then, after rinsing in PB, they were processed *via* the peroxidase antiperoxidase (PAP) method (Sternberger, 1979). The sections were incubated for 1 h in PB with 0.5% Triton-X and 3% BSA (Bovine Serum Albumin; Merck-Millipore, Darmstadt, Germany, A4503) and were then incubated for 24 h at 4°C in the same solution with the goat anti-orexin A (OXA; 1:500; Santa Cruz Biotechnology, Santa Cruz, CA, United States; sc-8070) or goat anti-orexin B (1:500; Santa Cruz Biotechnology; sc-8071). The specificity of the antisera used in the present study has been previously reported (Nixon and Smale, 2007; López et al., 2014; Lozano et al., 2018). The following day, the sections were rinsed three times in PB for 10 min and incubated for 1 h in rabbit anti-goat serum (1:50; Merck-Millipore, Darmstadt, Germany). Sections were then rinsed three times and incubated for 90 min in goat PAP complex (1:500; Merck-Millipore). Finally, the sections were rinsed three times in PB (for 10 min each time). They were then incubated in 0.5 mg/ml 3,3-diaminobenzidine and nickel with 0.01% H_2_O_2_ in PB (kit DAB; Vector Laboratories, Newark, CA, United States, SK4100). The sections were mounted on slides with 0.25% gelatin in 0.1M Tris-HCl buffer (pH 7.6), dehydrated and coverslipped with Entellan (Merck-Millipore).

### Immunofluorescence experiments

For immunofluorescence labeling procedures, the brain of each animal was removed and post-fixed by immersion in the same fixative for 24 h at 4°C. After rinsing in PB, the brains were cut in the coronal plane using a vibratome (Leica Biosystems, Barcelona, Spain; VT2100S). Serial sections (50 μm thick) were cryoprotected in 30% sucrose solution in 0.1 M PB and stored in ethyleneglycol/glycerol at −20°C until they were used. For immunofluorescence, two series of free-floating sections, selected at mid-anteroposterior levels of the hypothalamus, were rinsed thoroughly in 0.1 M PB and incubated for 1 h in 0.1 M PB with 0.25% Triton-X100 and 3% BSA. They were then incubated for 48 h at 4°C in the same blocking solution containing one of the following two combinations of primary antibodies: one set of sections was incubated with goat anti-OXA (1:500; Santa Cruz Biotechnology; sc-8070) and mouse anti-tyrosine hydroxylase (TH; 1:1,000; Immunostar, Hudson, WI, United States; P22941), rabbit anti-TH (1:1,000; Merck-Millipore; AB152) or rabbit anti-serotonin (5-HT; diluted 1:1,000; Immunostar, United States; 20080) antibodies. After rinsing in PB, the sections were incubated for 2 h at room temperature in a mixture of donkey anti-goat Alexa 594 (1:300; Invitrogen, Thermo Fisher Scientific, Waltham, MA, United States) with chicken anti-mouse Alexa 488 (1:300; Invitrogen) or with FITC- conjugated chicken anti-rabbit (1:100; Merck-Millipore; AP169F) antibodies.

The second set of sections was incubated with mouse anti-GM-130 (1:50; BD Biosciences, Eysins, Vaud, Switzerland; 610823), rabbit anti-C-Fos (1:2,000; Synaptic Systems GmbH, Goettingen, Germany; 226008) and goat anti-OXA (1:500; Santa Cruz Biotechnology; sc-8070) antibodies. After rinsing in PB, the sections were incubated for 2 h at room temperature in a mixture of donkey anti-mouse Alexa 647, donkey anti-rabbit Alexa 594 and donkey anti-goat Alexa 488 secondary antibodies (1:1,000; Molecular Probes).

In all cases, after rinsing in PB, the sections were also stained with the nuclear stain DAPI (4,6 diamino-2-phenylindole; Sigma, St. Louis, MO, United States). Finally, the sections were mounted in antifade mounting medium (ProLong Gold, Invitrogen) and studied by conventional fluorescence (Olympus BX51; Zeiss, 710) or confocal microscopy (Zeiss, 710). Conventional fluorescence tile images of the lateral hypothalamic area (20×) were used to quantify the intensity of C-Fos and OXA immunostaining in C-Fos/OXA/GM-130 triple-stained sections. Optical density measurements were taken with a polygonal sampling tool from the nucleus (C-Fos immunostaining channel) or the cytoplasm (OXA immunostaining channel) of every orexinergic neuron in sections from the control group (*n* = 177 neurons from four animals), arousal group (*n* = 288 neurons from five animals) and torpor group (*n* = 201 neurons from four animals), using Fiji software (Schindelin et al., 2012; open-source platform based on Image J^1^). The values obtained were normalized by subtracting the optical density value obtained after averaging at least ten measurements in the appropriate immunofluorescence channel from neuropil regions of the lateral hypothalamic area.

Additionally, confocal image stacks (30–150 images) from orexinergic cells of the lateral hypothalamic region were recorded from OXA/GM-130 double-stained sections, at 0.14 mm intervals through separate channels with a 63× oil-immersion lens (NA, 1.40, refraction index, 1.45) at zoom 2.0. Each immunofluorescence of OXA and GM-130 was automatically enhanced, after which single-pixel background fluorescence was eliminated by de-speckling. Colocalization of immunofluorescence for OXA and GM-130 was studied with the aid of Fiji software (JACoP tool) estimating the Manders coefficient (Manders et al., 1993) in cropped confocal stacks including complete single neurons (20 neurons per experimental group). Fiji software (3D Object counter) was used to analyze the volume of the Golgi complex elements immunostained for GM-130 in image stacks. We used ZEN 2012 software (Zeiss) to construct the maximum intensity projection images from the optical series by combining the images recorded through the different channels (image resolution: 1,024 × 1,024 pixels; pixel size: 0.066 mm). GraphPad Prism software (version 5.0) was used to create graphs and Adobe Photoshop (CS6, Adobe Systems, San José, CA, United States) software was used to compose figures.

SPSS software (IBM SPSS Statistics v25, IBM Corp., United States) was used for correlation analysis and to compare C-Fos and OXA immunolabeling values between groups (ANOVA test with Bonferroni *post hoc* comparisons). Kruskal-Wallis test with Dunn *post hoc* comparisons was used to compare Golgi apparatus (GA) mean volume values and Manders colocalization coefficient between experimental groups (the normality and homoscedasticity criteria were not met) with the aid of GraphPad Prism software (Prism 8.00 for Windows, GraphPad Software Inc., San Diego, CA, United States). Frequency distribution analysis of intensity of C-Fos and OXA immunostaining was performed using Kolmogorov-Smirnov (KS) non-parametric test (GraphPad Prism software, Prism 9.00 for Windows, GraphPad Software Inc., San Diego, CA, United States).

## Results

In the present work, the distribution pattern of orexin A- and orexin B-immunoreactive (-ir) cell bodies and fibers were studied in the brain of *M. auratus* using bright field immunohistochemical techniques. The neuroanatomical interactions between the orexinergic and the monoaminergic systems have also been analyzed by double immunofluorescence labeling. We followed the atlas of Morin and Wood (2001) as a reference to determine the borders between regions and nuclei. We also indirectly examined the activation of orexinergic neurons in the different phases of the hibernation cycle of the Syrian hamster by measuring the changes in the patterns of immunostaining in orexinergic neurons of the activity marker C-Fos, and of OXA in relation to the morphological changes of the Golgi apparatus.

### Orexinergic system in the brain of *Mesocricetus auratus*

**FIGURE 1:**
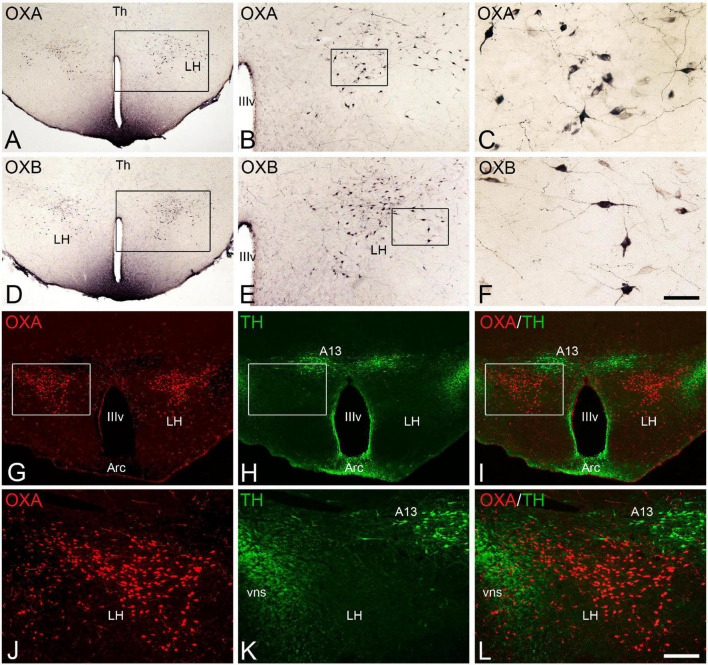
**(A–F)** Photomicrographs of transverse sections through the hypothalamus of *Mesocricetus auratus* showing the distribution of orexinergic neurons that expressed the neuropeptides orexin A [OXA **(A–C)**] or orexin B [OXB **(D–F)**] in the lateral hypothalamic area. Squared areas in panels **(A,B,D,E)** are respectively shown at higher magnification in panels **(B,C,E,F)**. **(G,H)** Pair of fluorescence photomicrographs from an OXA/TH double-immunostained section through the hypothalamus of *M. auratus* showing the distribution of orexinergic neurons (red) and TH-ir catecholaminergic neurons (green). **(I)** Merged image generated from panels **(G,H)**. Squared areas in panels **(G–I)** are shown at higher magnification in panels **(J–L)**. A13, A13 catecholaminergic cell group or zona incerta; Arc, arcuate nucleus; LH, lateral hypothalamus; Th, thalamus; vns, fibers of the nigrostriatal pathway; IIIv, third ventricle. Scale bar, shown in panel **(F)**, indicates 530 μm in panels **(A,D)**, 210 μm in panels **(B,E)**, and 50 μm in panels **(C,F)**. Scale bar, shown in panel **(L)**, indicates 200 μm in panels **(G–I)**, and 100 μm in panels **(J–L)**.

We examined the distribution of orexinergic neurons and fibers in the brain of euthermic hamsters using specific antibodies directed to orexin A or to orexin B (Figures 1A–F). The patterns of immunoreactivity revealed by both antibodies used in the present study were similar, and only some differences in the density of fiber innervation were observed in some brain regions (see below).

The orexinergic neurons (orexin A-ir and orexin B-ir) in the Syrian hamster form a conspicuous cell population restrictedly located in the lateral hypothalamus (Figure 1). These neurons, of medium size and generally with two or three long processes (Figures 1C,F), were observed in the dorsal aspect of the lateral hypothalamic area (Figures 1A,D,G) with a rostrocaudal extent of approximately 1,000 μm from postchiasmatic to premamillary hypothalamic levels. At the level of the prominent dopaminergic cell group of the zona incerta (A13), the orexinergic neurons were very numerous and were situated in the dorsolateral hypothalamus, ventrolaterally to A13 neurons, with these two cell populations clearly separated (Figures 1G–L). In contrast to orexin-ir cell bodies, orexinergic fibers were observed throughout the brain according to previous studies (Nixon and Smale, 2007), while the brainstem and the proper hypothalamus were the most densely innervated regions. Specifically, the tuberomamillary hypothalamic nucleus, the locus coeruleus, the dorsal raphe nucleus and the dorsal part of the solitary tract nucleus were the brain areas more densely innervated by orexin fibers, together with the paraventricular thalamic nucleus within the diencephalon. The orexin B-ir fibers were generally less dense than the orexin A-ir innervation in some areas such as the dorsal part of the lateral septum, the preoptic area, the supraoptic nucleus, the medial habenular nucleus, the inferior colliculus, or the dorsal motor nucleus of the vagus nerve and the ambiguous nucleus, according to previous studies (Nixon and Smale, 2007).

We then studied the neuroanatomical interactions between orexinergic fibers and the monoaminergic systems in the brain of euthermic animals using double-immunostained sections for OXA and 5-HT or TH (the first and rate-limiting enzyme in the synthesis of all catecholamines).

**FIGURE 2:**
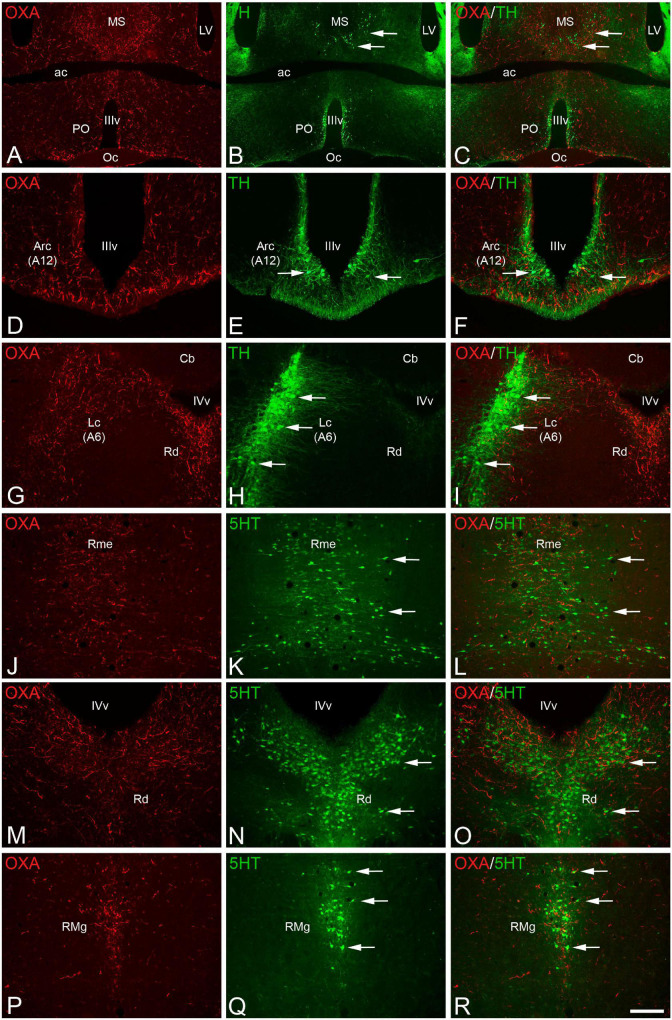
**(A,B,D,E,G,H,J,K,M,N,P,Q)** Pair of fluorescence photomicrographs from OXA/TH **(A–I)** or OXA/5HT **(J–R)** double-immunostained sections through different antero-posterior levels of the brain of *Mesocricetus auratus* showing the overlapping of orexinergic fibers (red fluorescence) and catecholaminergic **(A–I)** or serotonergic **(J–R)** neurons (arrows). **(C,F,I,L,O,R)** Merged images. Scale bar, shown in panel **(R)**, indicates 1,225 μm in panels **(A–C)** and 385 in panels **(D–R)**. ac, anterior commissure; Arc, arcuate nucleus; A6, noradrenergic cell group of locus coeruleus; A12, dopaminergic cell group of arcuate nucleus; Cb, cerebellum; Lc, locus coeruleus; LV, lateral ventricle; MS, medial septum; Oc, optic chiasm; PO, preoptic area; Rme, median raphe nucleus; Rd, dorsal raphe nucleus; RMg, magnus raphe nucleus; IIIv, third ventricle; IVv, fourth ventricle.

Starting with the catecholaminergic system, we observed that the dopaminergic telencephalic cells in the olfactory bulbs were sparsely innervated with orexin fibers, whereas this innervation was more intense in the TH-ir cells in the medial septum and preoptic area (Figures 2A–C). The dopaminergic cell populations within the hypothalamus (A11 to A15 groups) were generally well-innervated with orexinergic fibers, and this innervation was particularly abundant in the paraventricular and arcuate nuclei (Figures 2D–F). Similarly, the mesencephalic dopaminergic groups showed abundant orexin innervation, especially in the ventral tegmental area (A10), and less markedly in the *substantia nigra pars compacta* (A9) and retrorubral area (A8). In the rostral rhombencephalon, the noradrenergic cell population most intensely innervated with orexinergic fibers was the locus coeruleus (A6; Figures 2G–I), although groups A4, A5, and A7 also showed a remarkable orexinergic innervation. Finally, in the caudal rhombencephalon, the mixture of noradrenergic and adrenergic cells of A2/C2 and A3/C3 groups surrounding the solitary tract were strongly innervated with orexin fibers.

The interactions between the orexinergic and serotonergic systems were revealed by double immunolabeling for orexin A and serotonin. The whole raphe column was remarkably innervated by orexinergic fibers, but this innervation was especially profuse in the median (Figures 2J–L) and dorsal (Figures 2M–O) raphe nuclei within the rostral portion of the column, and in the magnus (Figures 2P–R) and pallidus raphe nuclei in the caudal part of the column. In clear contrast with the medium to high levels of orexinergic innervation in the monoaminergic groups described above, catecholaminergic and serotonergic innervation in the orexinergic cells in the lateral hypothalamus is relatively low (Figures 1K,L).

### C-Fos expression in orexinergic neurons during hibernation

**FIGURE 3:**
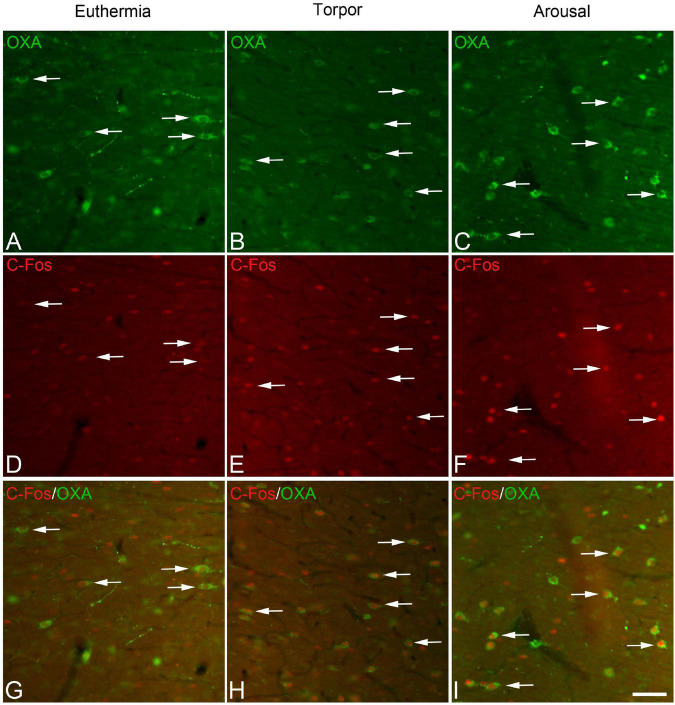
Pairs **(A/D, B/E, C/F)** of fluorescence photomicrographs of OXA/C-Fos double-immunostained sections from the LH of *Mesocricetus auratus* showing the expression of the neuronal activity marker C-Fos (red) in orexinergic neurons (arrows) of hamsters in euthermic, arousal, and torpor states. **(G,H)** and **(I)** show merged images of **(A/D, B/E, **C/F**)**, respectively. Note the intense C-Fos labeling of LH in hamsters at arousal **(F)**. Scale bar, shown in panel **(I)**, indicates 60 μm.

The expression of the neuronal activity marker C-Fos was subsequently studied in orexinergic neurons in each of the hibernation phases using C-Fos/OXA/GM-130 triple-immunostained sections. We initially observed—by conventional fluorescence microscopy— that C-Fos immunostaining in the lateral hypothalamus, likely including orexinergic neurons, was apparently more intense in hamsters at arousal than in those in euthermia or torpor states (Figure 3).

**FIGURE 4:**
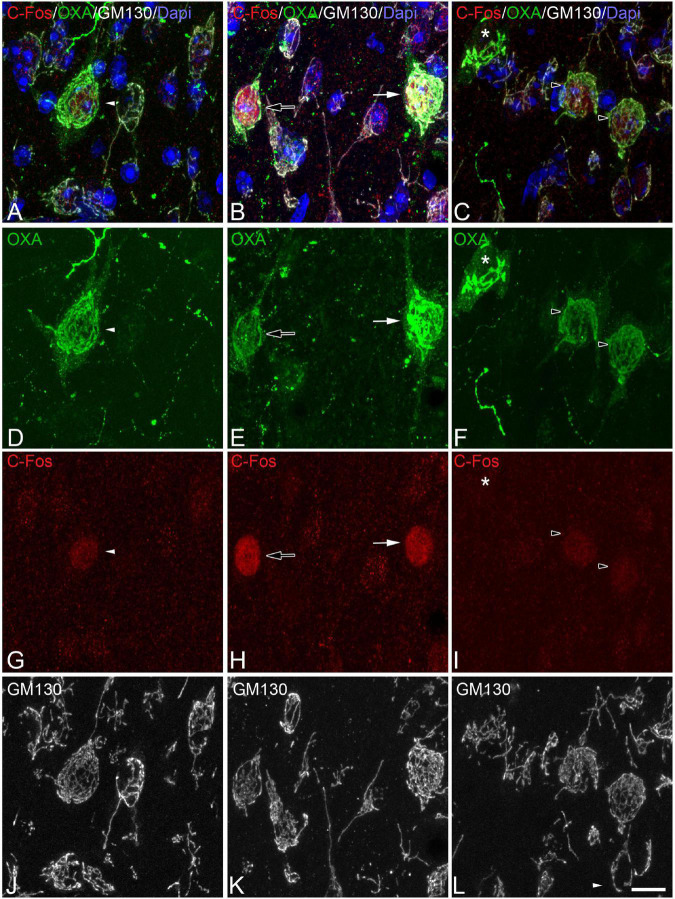
**(A-D-G-J, B-E-H-K, C-F-I-L)** Groups of confocal projection images taken from OXA/C-Fos/GM130 triple-immunostained sections and counterstained with DAPI, showing examples of LH orexinergic neurons, from euthermic Syrian hamsters, with different patterns of immunostaining. Note the presence of orexinergic neurons with intense OXA immunostaining showing intense [solid arrows in panels **(B,E,H)**] or moderate [solid arrowheads in panels **(A,D,G)**] C-Fos expression—or with no C-Fos immunostaining [asterisks in panels **(C,F,I)**]. Note also the presence of orexinergic neurons with moderate OXA immunostaining and intense [hollow arrows in panels **(B,E,H)**] or moderate [hollow arrowheads in panels **(C,F,I)**] C-Fos expression. Note also both orexinergic and non-orexinergic LH neurons in euthermic hamsters showing normal patterns (see text) of the GA, as revealed by GM130 immunostaining. Scale bar shown in panel **(L)** indicates 10.5 μm.

**FIGURE 5:**
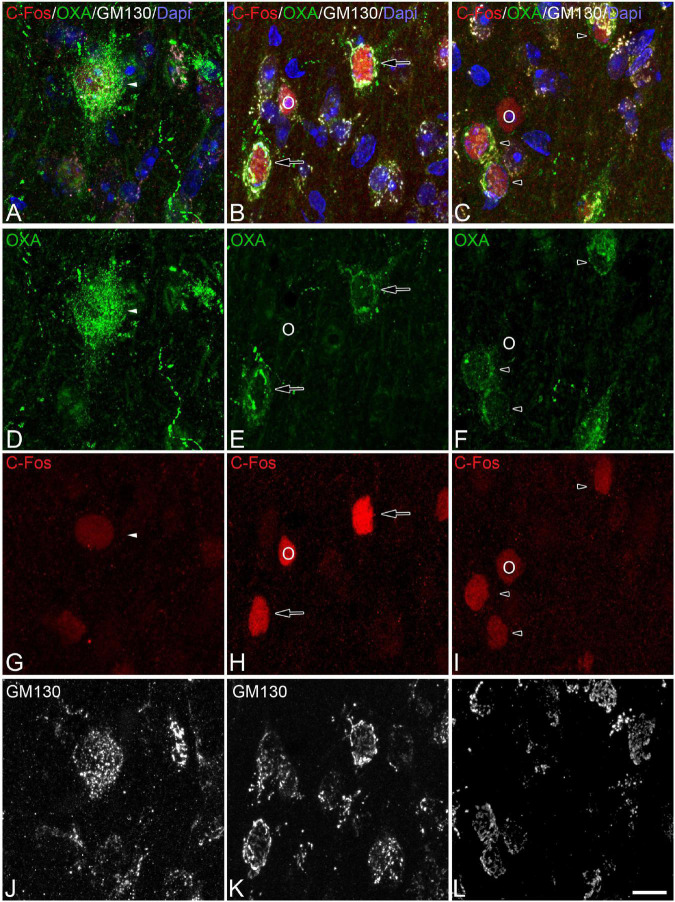
**(A-D-G-J, B-E-H-K, C-F-I-L)** Groups of confocal projection images taken from OXA/C-Fos/GM130 triple-immunostained sections and counterstained with DAPI, showing examples of LH orexinergic neurons, from Syrian hamsters at torpor, with different patterns of immunostaining. Solid arrowheads in panels **(A,D,G)** point to an orexinergic neuron with intense OXA immunostaining showing moderate C-Fos expression. Note the presence of orexinergic neurons with moderate OXA immunostaining showing intense [hollow arrows in panels **(B,E,H)**] or moderate [hollow arrowheads in panels **(C,F,I)**] C-Fos expression. The O symbols indicate examples of non-orexinergic neurons with intense **(B,E,H)** or moderate **(C,F,I)** C-Fos expression. Also note both orexinergic and non-orexinergic LH neurons in torpid hamsters showing altered (fragmented) patterns (see text) of the GA, as revealed by GM130 immunostaining. Scale bar shown in panel **(L)** indicates 10.5 μm.

**FIGURE 6:**
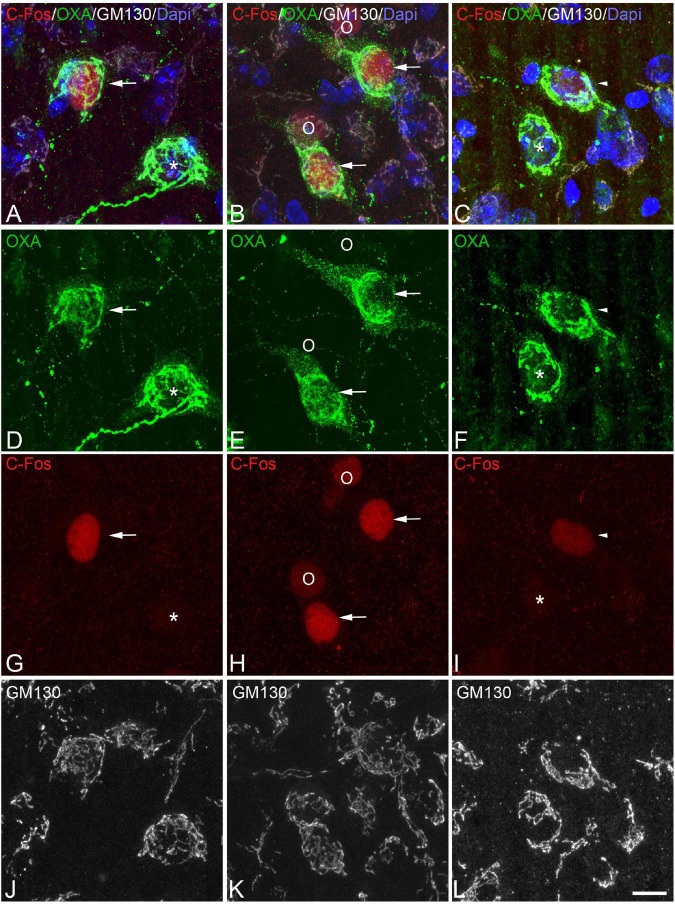
**(A-D-G-J, B-E-H-K, C-F-I-L)** Groups of confocal projection images taken from OXA/C-Fos/GM130 triple-immunostained sections and counterstained with DAPI, showing examples of LH orexinergic neurons, from Syrian hamsters at arousal, with different patterns of immunostaining. Note the presence of orexinergic neurons with intense OXA immunostaining showing intense [solid arrows in panels **(A,B,D,E,G,H)**] or moderate [solid arrowheads in panels **(C,F,I)**] C-Fos expression—or with no C-Fos immunostaining [asterisks in panels **(A,D,G,C,F,I)**]. The O symbols in panels **(B,E,H)** indicate examples of non-orexinergic neurons with moderate C-Fos expression. Also note both orexinergic and non-orexinergic LH neurons in hamsters at arousal showing patterns of the GA similar to those in euthermic animals. Scale bar shown in panel **(L)** indicates 10.5 μm.

**FIGURE 7:**
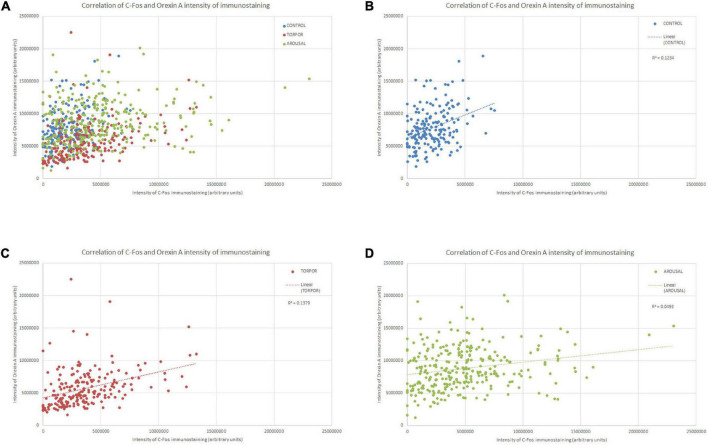
Diagrams showing the correlation between the intensity of C-Fos (X axis) and OXA (Y axis) immunostainings of all orexinergic neurons analyzed **(A)** and those analyzed from hamsters at euthermia **(B)**, torpor **(C)**, and arousal **(D)**. See Table 1 for R, R2, and *p*-values.

**FIGURE 8:**
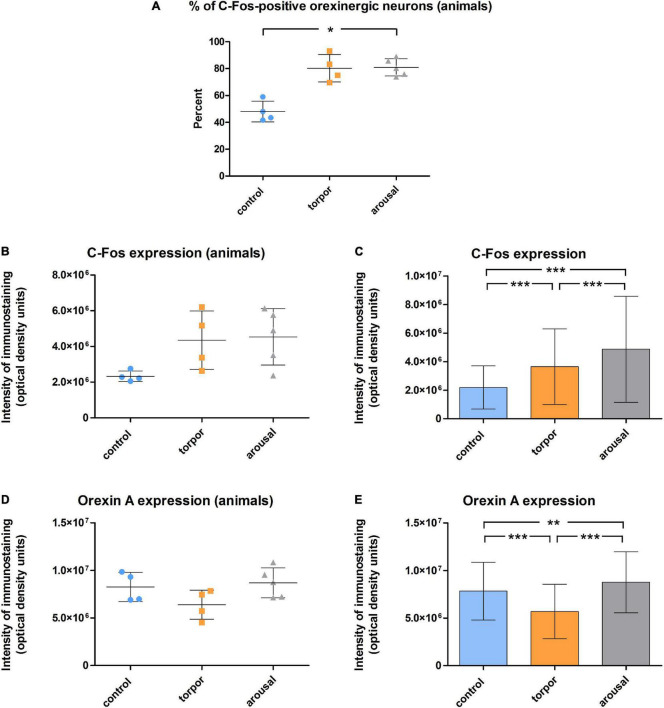
**(A)** Graph showing the mean ± SD values of the percentage of orexinergic neurons that showed C-Fos immunostaining in hamsters at euthermia (*n* = 4), torpor (*n* = 4), and arousal (*n* = 5). **(B–E)** Graphs showing mean ± SD values of C-Fos **(B)** and OXA **(D)** immunostaining of orexinergic neurons. Panels **(C,E)** show respectively mean ± SD values of C-Fos and OXA immunostaining of orexinergic neurons when all C-Fos-ir orexinergic neurons from the animals in each experimental condition were considered together (control, *n* = 177; torpor, *n* = 201; arousal, *n* = 288). See Tables 2, 3 for statistical comparisons and *p*-values. *p ≤ 0.05, **p ≤ 0.005, ***p ≤ 0.0001.

**FIGURE 9:**
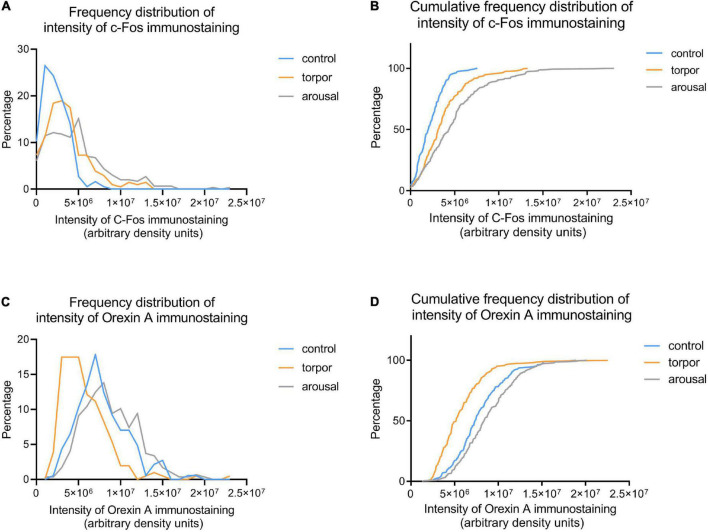
Frequency **(A,C)** and cumulative frequency **(B,D)** plots showing the intensity of C-Fos **(A,B)** and OXA **(C,D)** immunostaining of orexinergic neurons in hamsters at euthermia (blue), torpor (orange), and arousal (gray). See Table 4 for Kolmogorov-Smirnov D and *p*-values.

In order to gain insight in this preliminary observation and to understand how the orexinergic activity may change during hibernation, we next analyzed C-Fos and OXA immunostaining of orexinergic neurons in hamsters at euthermia, torpor, and arousal (Figures 4–6). We found that there is great heterogeneity in the intensity of C-Fos and OXA immunostaining of orexinergic neurons regardless of the hibernation state. In euthermic hamsters, orexinergic cells with low, moderate or high intensity of OXA immunostaining showing different levels of C-Fos expression were found (Figure 4). Similar heterogeneity was also observed in hamsters at torpor (Figure 5) and arousal (Figure 6). Using densitometry, we quantified the intensity of OXA and C-Fos immunostaining in the cytoplasm and nucleus, respectively, in orexinergic neurons from the control group (*n* = 177 neurons from four animals), arousal group (*n* = 288 cells from five animals) and torpor group (*n* = 201 cells from four animals) (Figures 7–9). Correlation analysis showed a weak to moderate—but statistically significant—positive correlation between the intensity of C-Fos and OXA immunostainings considering all of the orexinergic neurons together (Figure 7A and Table 1). This indicated that the intensity of OXA expression might, at least partially, depend on the neuronal activity indicated by C-Fos accumulation. Similar correlations were found when we only considered orexinergic neurons from euthermic hamsters (Figure 7B), and hamsters at the torpor (Figure 7C) and arousal hibernation phases (Figure 7D).

**TABLE 1:** R, R^2^, and *p*-values corresponding to the correlation analysis (SPSS software, v.22) between the intensity of C-Fos and OXA immunostainings of all orexinergic neurons analyzed, and those analyzed from hamsters at euthermia, torpor, and arousal.

	R	R^2^	*P*	*n*
All neurons	0.281	0.0795	0.000	666
Control	0.359	0.1234	0.000	177
Torpor	0.373	0.1379	0.000	201
Arousal	0.212	0.0493	0.000	288

We found significant differences (*p* = 0.02) in the percentage of orexinergic neurons showing levels of C-Fos immunostaining above background levels between animals in the different experimental conditions (Figure 8A). These differences were significant between hamsters at euthermia and arousal (Kruskal-Wallis with Dunn *post hoc* comparisons; see Table 2 for mean ± SD and *p*-values). When we compared mean values of C-Fos and OXA immunostaining of C-Fos-ir orexinergic neurons in hamsters at euthermia, torpor, and arousal (Figures 8B,D), we found no statistically significant differences (Kruskal-Wallis) between animals in the different experimental conditions, probably due to the large variability in immunostaining levels of orexinergic neurons (see below). However, when all orexinergic neurons from the animals in each experimental condition were considered together (Figures 8C,E), we found significant differences in C-Fos and OXA immunostaining of orexinergic neurons for control, torpor, and arousal conditions (ANOVA with Bonferroni *post hoc* comparisons; see Table 3 for *p*-values).

**TABLE 2:** Table showing mean ± SD values of the percentage of orexinergic neurons positive for C-Fos from hamsters at euthermia, torpor, and arousal; *p*-values corresponding to the comparisons between total cells obtained from hamsters in the different hibernation phases (Kruskal-Wallis with Dunn; SPSS software, v.22).

Intensity of C-Fos immunostaining			

Experimental groups	Mean ± SD	Comparisons between groups	*P*-values
% of orexinergic neurons (*n* = 442) immunoreactive for C-Fos-ir from euthermic hamsters	48.02 ± 7.75	Control vs. Torpor	ns
% of orexinergic neurons (*n* = 250) immunoreactive for C-Fos-ir from hamsters at torpor	80.27 ± 10.24	Control vs. Arousal	*
% of orexinergic neurons (*n* = 356) immunoreactive for C-Fos-ir from hamsters at arousal	80.98 ± 6.40	Torpor vs. Arousal	ns

*p ≤ 0.05.

**TABLE 3:** Table showing mean ± SD values of C-Fos (top) and orexin A (bottom) intensity of immunostaining of orexinergic neurons from hamsters at euthermia, torpor, and arousal; *p*-values corresponding to the comparisons between total cells obtained from hamsters in the different hibernation phases (ANOVA with Bonferroni; SPSS software, v.22).

Experimental groups	Mean ± SD	Comparisons between groups	*P*-values
**Intensity of C-Fos immunostaining**			
OXA-ir neurons (*n* = 177) from euthermic hamsters	2.29 ± 1.47 × 10^6^	Control vs. Torpor	***
OXA-ir neurons (*n* = 201) from hamsters at torpor	3.73 ± 2.62 × 10^6^	Control vs. Arousal	***
OXA-ir neurons (*n* = 288) from hamsters at arousal	5.02 ± 3.67 × 10^6^	Torpor vs. Arousal	***
**Intensity of OXA immunostaining**			
OXA-ir neurons (*n* = 177) from euthermic hamsters	7.86 ± 3.06 × 10^6^	Control vs. Torpor	***
OXA-ir neurons (*n* = 201) from hamsters at torpor	5.73 ± 2.83 × 10^6^	Control vs. Arousal	**
OXA-ir neurons (*n* = 288) from hamsters at arousal	8.83 ± 3.18 × 10^6^	Torpor vs. Arousal	***

**p ≤ 0.005, ***p ≤ 0.0001.

Frequency distribution analysis confirmed the significant differences between the distribution of C-Fos and OXA intensity of immunostaining values for orexinergic cells obtained from animals at different phases of hibernation (Figure 9 and Table 4). The results showed that, as compared with values at euthermia, there was a significant increase in the mean intensity of C-Fos immunostaining of orexinergic neurons during torpor, due to an increase in the percentage of orexinergic neurons with elevated C-Fos expression levels (note the right shifts of the curves in Figures 9A,B). This increase in C-Fos immunostaining intensity at torpor was paralleled by a decrease in the expression of OXA (Figures 8, 9). In animals at arousal, there was a significant increase in the percentage of orexinergic neurons with increased intensity of C-Fos and OXA immunostaining, with statistically significant differences for orexinergic neurons from both euthermic and torpid animals (Figures 8, 9).

**TABLE 4:** Table showing D and *p*-values corresponding to the comparison between control, torpor, and arousal experimental groups of the frequency distribution of C-Fos (top) and OXA (bottom) immunostaining of orexinergic cells (Kolmogorov-Smirnov; GraphPad Prism software, v.9).

Comparisons between groups	KS D values	*P*-values
**Intensity of C-Fos immunostaining**		
Control vs. Torpor	0.2815	***
Control vs. Arousal	0.4294	***
Torpor vs. Arousal	0.2209	***
**Intensity of OXA immunostaining**		
Control vs. Torpor	0.3765	***
Control vs. Arousal	0.1663	**
Torpor vs. Arousal	0.4471	***

**p ≤ 0.005, ***p ≤ 0.0001.

### Changes in the Golgi apparatus of orexinergic neurons during hibernation

To study the possible modifications of the GA of LH orexinergic neurons during the three hibernation phases of the Syrian hamster, we analyzed—by confocal microscopy and 3D quantification techniques in C-Fos/OXA/GM-130 triple-immunostained sections—the changes in the expression patterns of the GA marker GM-130, including the volume of GA GM-130-ir elements and the OXA/GM-130 Manders colocalization coefficient (Manders et al., 1993).

**FIGURE 10:**
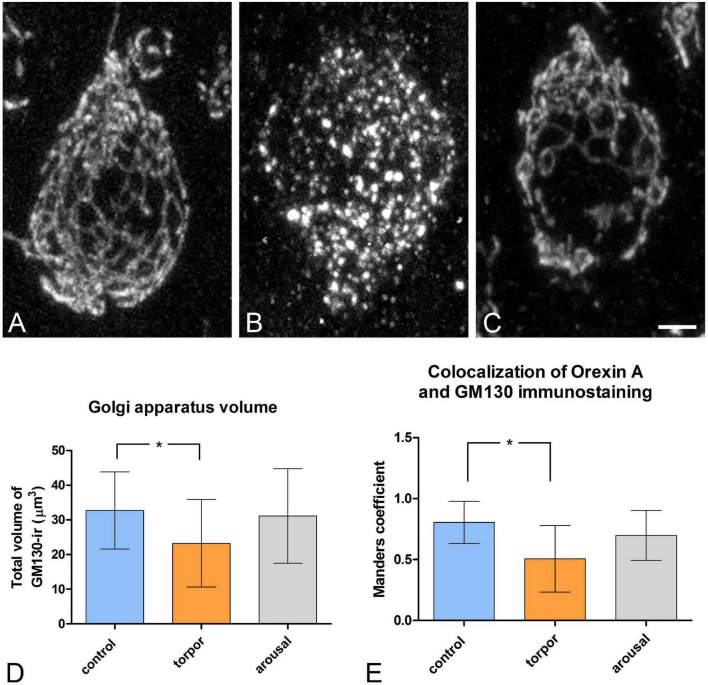
Distribution of the GA protein GM-130 in LH orexinergic neurons of Syrian hamsters in the three phases of hibernation. **(A–C)** Confocal Z-projected images showing GM-130-immunostaining of orexinergic cells, obtained from OXA/C-Fos/GM130 triple-immunostained sections from hamsters at euthermia **(A)**, torpor **(B)**, and arousal **(C)**. Note the fragmentation of the GA during torpor and its recovery during arousal. Scale bar indicates 3 μm. Histograms showing mean ± SD values of volume of the GM130-ir GA elements **(D)**, and of OXA/GM130 Manders colocalization coefficient **(E)** obtained from cropped confocal image stacks of complete orexinergic neuronal somata from control, arousal, and torpor animals (*n* = 20 for each experimental condition). Kruskal-Wallis and Dunn *post hoc* tests found significant differences in both GA volume and Manders coefficient mean values between control and torpor groups (p* ≤ 0.05).

Despite the apparent lack of changes in the intensity of GM-130 immunostaining in orexinergic neurons between animals in the different phases of hibernation, the appearance of GM-130 immunostaining was different in euthermic animals as compared to animals in the torpor and arousal phases (Figures 4–6, 10). In euthermic animals, the appearance of the GA in LH orexinergic neurons consisted of a network of twisted and convoluted cisternae and tubular structures with a ribbon-like appearance distributed through the cytoplasm surrounding the nucleus, extending to the main dendritic processes (Figures 4, 10A). However, in all neurons analyzed from torpid animals, the GA showed a drastically different morphology with a peculiar granular and fragmented appearance (Figures 5, 10B). However, the GA elements of orexinergic neurons from animals at arousal showed a similar morphology to that of euthermic animals, although they occasionally showed a small and limited fragmentation (Figures 6, 10C).

We quantified the total volume of GM-130-ir GA elements in confocal image tiles using the 3D object counter tool in the Fiji software package. We found variations in the volume of GM-130- ir elements in the different hibernation phases, with a statistically significant decrease during torpor, as compared to control (p* = 0.0155) (Figure 10D). During arousal, the mean volume of GM-130-ir GA elements of orexinergic neurons showed intermediate values, but with no significant differences, as compared to the values for euthermic and torpid hamsters (Figure 10D).

To gain a deeper insight into the distribution patterns of OXA in euthermic hamsters and their possible changes during hibernation, we next quantified—using the JACoP tool in the Fiji software package—the degree of OXA/GM-130 colocalization in LH orexinergic neurons from animals at the three phases of hibernation (Figure 10E). In euthermic animals, we observed a mean Manders coefficient of 0.804, indicating that around 80% of the voxels positive for OXA were also positive for GM-130. The fragmentation and the decrease in the volume of the GA observed in hamsters at torpor were accompanied by a statistically significant (*p* = 0.0065) decrease in the OXA/GM-130 Manders colocalization coefficient during torpor (M1 = 0.505). At arousal, the orexinergic neurons showed intermediate OXA/GM-130 Manders colocalization coefficient values, but with no significant differences, as compared to those from euthermic and torpid hamsters (Figure 10E).

## Discussion

In the present study, the organization of the orexinergic system in the brain of Syrian hamsters and the possible interactions with the monoaminergic systems were investigated using immunohistochemical techniques. In addition, changes that orexinergic neurons undergo throughout the different phases of the hibernation cycle were analyzed by confocal microscopy and 3D quantitative techniques. Our main findings were the existence of a close neuroanatomical relationship between the orexinergic system and the catecholaminergic and serotonergic systems, as well as the possible participation of orexins in the regulation of physiological events that occur in the different stages of hibernation.

### Distribution of orexinergic neurons in the brain of *Mesocricetus auratus*

The structure of orexins is very similar in all vertebrates, with only some variations in the sequence of amino acids. The C-terminal sequence is remarkably constant in the orexin peptides of all the vertebrate species examined (Sakurai et al., 1998; Shibahara et al., 1999; Wong et al., 2011). The antisera anti-OXA and anti-OXB used in the present study were raised against a peptide mapping at the C-terminus of human OXA or OXB. This would explain why there were no significant differences in the pattern of immunolabeling obtained with both antibodies in the present and previous studies (Singletary et al., 2006; López et al., 2009a,b, 2014, 2016; Domínguez et al., 2010; Miranda et al., 2013; Lozano et al., 2018), beyond the finding of an apparently less dense distribution of OXB-ir fibers as compared to OXA-ir fibers in some brain regions such as the dorsal part of the lateral septum, the preoptic area, the supraoptic nucleus, the medial habenular nucleus, the inferior colliculus, or the dorsal motor nucleus of the vagus nerve and the ambigous nucleus, among others—in agreement with previous observations (Nixon and Smale, 2007).

In the present study, we found that the cell bodies of orexinergic neurons in the Syrian hamster were located restrictedly in the dorsal aspect of the lateral hypothalamus from postchiasmatic to premamillary hypothalamic levels, in agreement with previous studies using different antibodies (McGranaghan and Piggins, 2001; Mintz et al., 2001; Nixon and Smale, 2007). In other rodent species such as lab rats, grass rats and degus, the presence of orexinergic neurons is more extensive and they have been localized in additional hypothalamic regions including the dorsomedial hypothalamus and the perifornical, supraoptic and paraventricular nuclei (Nixon and Smale, 2007). In addition, a group of orexinergic cells has been reported in the ventrolateral hypothalamus of megachiropteran species, adjacent to the optic tract (Kruger et al., 2010; Dell et al., 2013), and exclusively in the medial hypothalamus of the giraffe and harbor porpoise (Dell et al., 2012). Despite these interspecies variations in the precise location of orexinergic cells, their presence in the basal hypothalamus and their interactions with monoaminergic nuclei seem to be a primitive and conserved feature shared in all mammalian and non-mammalian vertebrates (Kaslin et al., 2004; Huesa et al., 2005; Suzuki et al., 2008; López et al., 2009a,b, 2014; Domínguez et al., 2010; Lozano et al., 2018). This suggests a conservation of their functions consisting of the integration of a variety of circadian, interoceptive and homeostatic signals such as hunger, stress, and autonomic challenges to increase behavioral arousal *via* actions on multiple neurotransmitter systems, such as the monoaminergic, cholinergic, and histaminergic systems (Teske et al., 2010; Sakurai and Mieda, 2011; Inutsuka and Yamanaka, 2013; Schwartz and Kilduff, 2015).

### Orexinergic projections with potential roles in the regulation of specific functions during hibernation

The widespread pattern of orexinergic fibers found in the Syrian hamster in the present study is in agreement with previous reports (Mintz et al., 2001; Nixon and Smale, 2007), and is similar to the pattern reported in other rodent species (Peyron et al., 1998; Nixon and Smale, 2007). Although electron microscopy has not been used in the present work—and would be necessary to demonstrate synaptic contacts—double immunofluorescence for OXA and TH, or 5-HT, revealed possible interactions between these systems in the brain of euthermic *M. auratus*. The orexinergic cell population in the lateral hypothalamus of the Syrian hamster was found to be innervated by catecholaminergic and serotonergic fibers, although apparently with a lower density to that in the rat (Kirchgessner, 2002; Lee H. S. et al., 2005), in which orexin neurons were found to be directly hyperpolarized by noradrenaline, dopamine, and serotonin (Yamanaka et al., 2003, 2006; Muraki et al., 2004) and, therefore, inhibited by these monoamines. The reciprocal connections were more clearly established in the Syrian hamster since, in addition to other brain regions (see below), striking orexinergic innervation of dopaminergic, noradrenergic and serotonergic cell groups was found, in agreement with previous studies in other rodents (Kirchgessner, 2002; Baldo et al., 2003; Ferguson and Samson, 2003; Bubser et al., 2005; Lee H. S. et al., 2005; Yoshida et al., 2006; Balcita-Pedicino and Sesack, 2007).

Orexins have been related to the regulation of energy metabolism-dependent food intake (feeding) behavior in non-hibernating animals (Kilduff and Peyron, 2000; Sutcliffe and de Lecea, 2002; Taheri et al., 2002; Khorooshi and Klingenspor, 2005; Sakurai, 2005; Kotz, 2006). Orexinergic neurons can function as glucose sensors, since extracellular glucose inhibits these neurons by opening their potassium channels, leading to cell hyperpolarization (Burdakov et al., 2006). Orexinergic cells modulate food intake through intrahypothalamic connections to the arcuate, paraventricular, and dorsomedial hypothalamic nuclei (Peyron et al., 1998; Date et al., 1999). In the arcuate nucleus, orexins stimulate food intake by activating neuropeptide Y (NPY)-releasing neurons and inhibiting proopiomelanocortin (POMC)-releasing neurons (Arrigoni et al., 2019). In addition, orexinergic neurons may also promote feeding due to innervation of the paraventricular hypothalamic nucleus, through which both POMC and NPY neurons regulate hunger and satiety (Arrigoni et al., 2019). In addition to feeding, OXA has been shown to participate in the maintenance of body temperature (Sakurai et al., 1998; Dube et al., 1999; Volkoff et al., 1999; Hara et al., 2001; Harris and Aston-Jones, 2006; Volkoff, 2006; Carter et al., 2009; Avolio et al., 2011; Girault et al., 2012; Milbank and López, 2019) and in energy expenditure on brown adipose tissue non-shivering thermogenesis (Madden et al., 2012; Zhang and Bi, 2015)—the main sympathetic nervous system-mediated mechanism to regain body temperature after hypothermia (Yasuda et al., 2005; Kotz, 2006). Previous studies have shown that OXA stimulation in various brain areas increases thermogenesis and energy expenditure *via* the activation of the sympathetic nervous system, affecting brown adipose tissue (BAT) and increasing heat production (Huang et al., 2001; Hitrec et al., 2020). The hypothalamic orexigenic projections to the raphe pallidus have been shown to increase cold–initiated BAT thermogenesis in the rat (Tupone et al., 2011). Conversely, a reduced orexin synthesis results in a deficit in heat production capacity, and it has been reported that orexins play a protective role against hypothermia induced by lack of food (Futatsuki et al., 2018). In hibernating mammals, feeding is abandoned and temperature is highly reduced during torpor. However, thermoregulation and feeding in some hibernator species including the Syrian hamster are reactivated during arousals between bouts of hibernation (Berger, 1984). In the present study, a remarkable orexinergic innervation was observed in the arcuate nucleus of the Syrian hamster (present results) in agreement with previous studies (Nixon and Smale, 2007), suggesting that feeding in hamsters could be regulated by orexin-mediated mechanisms, similar to those present in other rodent species. In fact, it has been shown in hibernating hamsters that orexins and the sympathoinhibitory neuroactive peptide catestatin differentially modulate orexin-2 receptor-dependent feeding and motor behaviors throughout the different hibernation phases (Mele and Canonaco, 2014; Mele et al., 2014) and that OXA activity favors feeding and motor behaviors during the arousal phase (Sakurai et al., 1998; Avolio et al., 2012; Mele et al., 2014).

Regarding sleep, accumulating evidence suggests the involvement of orexinergic projections to the tuberomamillary hypothalamus, to the locus coeruleus and to the raphe nuclear complex in the regulation of the sleep-wake cycle. Orexins depolarize histaminergic neurons of the tuberomamillary posterior hypothalamus that express OXR2 receptors and contribute to the maintenance of wakefulness (Trivedi et al., 1998; Eriksson et al., 2001; Huang et al., 2001; Ishizuka et al., 2002; Geerling et al., 2003; Yamanaka et al., 2003; Schone et al., 2014). In line with these studies, in the present work, we found prominent orexinergic innervation of the tuberomamillary region of the hypothalamus in the Syrian hamster. In addition, the noradrenergic cell groups in the upper rhombencephalon that constitute the locus coeruleus complex of *M. auratus* are richly innervated by orexin-ir fibers. Similar orexinergic projections were demonstrated in the locus coeruleus of the rat (Peyron et al., 1998; Baldo et al., 2003), whose neurons express the OX1R receptor (Marcus et al., 2001) and whose activation is crucial for the inhibition of REM sleep (Ohno and Sakurai, 2008). The orexinergic innervation activates the liberation of noradrenaline by the locus coeruleus (Hagan et al., 1999; Walling et al., 2004; Chen et al., 2008) and controls locus coeruleus-cerebrocortical noradrenergic activity (Tose et al., 2009). In addition, the orexinergic neurons projecting to the locus coeruleus show a circadian rhythm—they are generally quiescent during quiet wakefulness, slow wave (non-REM or SWS) and REM sleep but show high discharge rates during active wakefulness and in anticipation of REM sleep-to-wake transitions (Estabrooke et al., 2001; Lee M. G. et al., 2005; Mileykovskiy et al., 2005; Takahashi et al., 2008). Their activation, which correlates with the neuronal firing frequency of the locus coeruleus, is very important in the modulation of day-night differences of the locus coeruleus impulse activity that promote wakefulness and behavioral arousal (Gompf and Aston-Jones, 2008; del Cid-Pellitero and Garzon, 2011; Kohlmeier et al., 2013). Therefore, the connections between the orexinergic system and the locus coeruleus have been shown to be essential to promote the sleep-to-wake transitions and general arousal in non-hibernating mammalian species (de Lecea et al., 1998; Sakurai et al., 1998; Saper, 2006; Carter et al., 2012; de Lecea, 2015). It has also been shown that the sensitivity of orexin neurons to noradrenergic innervation (Grivel et al., 2005) and the efficiency of orexin neurons to induce awakenings depend on sleep pressure or deprivation [see de Lecea (2015)]. This provides a mechanism through which orexinergic cells sense previous sleep history.

The serotonergic cells of the raphe column of *M. auratus* are richly innervated by OX-ir fibers and terminal structures (present results), as has been observed in each subdivision of the raphe nuclei of non-hibernating rodents in previous studies (Wang et al., 2003; Lee H. S. et al., 2005). Raphe cells express OX1R and OX2R receptors (Marcus et al., 2001) and their activation depolarizes the dorsal raphe neurons (Kohlmeier et al., 2008) stimulating serotonin release (Takahashi et al., 2005; Wang et al., 2005; Tao et al., 2006; del Cid-Pellitero and Garzon, 2011; Adidharma et al., 2012), which inhibits both REM and non-REM sleep (Ohno and Sakurai, 2008). Thus, orexins in non-hibernating rodents play a regulatory role in brain functions under efferent regulation of raphe nuclei (Lee H. S. et al., 2005), including the control of the arousal and sleep-wakefulness cycle. The rich orexin innervation of the raphe nuclei in the Syrian hamster suggests that this may also occur in this species.

In hibernating mammals, hibernation bouts may only be initiated when the animal is asleep and they always begin with an SWS episode—and therefore hibernation was considered an extension of SWS sleep (Satinoff and Rutstein, 1970; Walker and Berger, 1980; Berger, 1984; Nedergaard and Cannon, 1990). As such, it was suggested that SWS, deep torpor during hibernation and daily torpor were homologous adaptations for energy conservation (Heller and Glotzbach, 1977; Heller et al., 1978). However, later studies reported that EEG during early phases of deep torpor might be different from the SWS EEG of euthermia (Deboer and Tobler, 1995). It was then proposed that sleep was among the important physiological processes that are downregulated during deep torpor bouts, as sleep seems to be incompatible with hypometabolic state and low brain temperature (Blanco et al., 2016). An absence of sleep during deep torpor bouts has been reported in non-primate hibernators (arctic golden-mantled and European ground squirrels) and in two species of hibernating lemurs (Daan et al., 1991; Trachsel et al., 1991; Strijkstra and Daan, 1997; Blanco et al., 2016). Therefore, it was proposed that deep torpor during hibernation with pronounced drops in body temperature was no longer considered a process homologous to sleep and daily or shallow torpor (Palchykova et al., 2002; Rial et al., 2010), which are characterized by only moderate reductions in body temperature (Berger, 1984). In fact, the restorative functions associated with SWS do not appear to occur during the hibernation torpor phase, perhaps due to the low body temperature [for a review, see Kilduff et al. (1993)]. It has been suggested that a slow-wave sleep debt accumulates during deep torpor periods of hibernation, when slow-wave sleep is inhibited. By contrast, thermogenically induced arousals, which are considered to play neuroprotective roles, would be needed to periodically fulfill the physiological critical demand of the restorative functions of sleep (Daan et al., 1991; Trachsel et al., 1991; Berger, 1993; Palchykova et al., 2002; Arendt et al., 2003; Blanco et al., 2016). During spontaneous arousals, in both non-primate and primate hibernators, including squirrels (Walker et al., 1977; Daan et al., 1991; Trachsel et al., 1991), echidnas (Allison and van Twyver, 1972) and lemurs (Blanco et al., 2016), there is a preponderance of sleep consisting of both REM and non-REM sleep, but with a predominance of SWS during early arousal and a subsequent decline in a gradual transition to the torpid EEG. The involvement of orexinergic neurons in the regulation of sleep patterns during the interbout arousal periods in hibernating mammals, although likely, has not yet been directly explored.

We also observed abundant orexinergic fibers and terminals interspersed with the adrenergic and noradrenergic neurons of the nucleus of the solitary tract. An equivalent orexinergic innervation, described also in non-hibernating rodents (Kirchgessner, 2002; Baldo et al., 2003; Ferguson and Samson, 2003), has been shown to activate both OX1R and OX2R receptors (Marcus et al., 2001), stimulating the liberation of noradrenaline by neurons of the solitary tract nucleus (Smith et al., 2002; Ciriello et al., 2003). This sequence of events is very likely the basis for the orexinergic regulation of autonomic-cardiovascular responses, such as heart rate and arterial pressure (Smith et al., 2002; Ciriello et al., 2003, 2013; de Oliveira et al., 2003; Shih and Chuang, 2007; Shahid et al., 2012).

### Changes in orexinergic neurons during hibernation

In non-hibernating mammalian species, orexinergic neurons act as the gatekeeper of behavioral state stability (Bonnavion et al., 2016). The activity of these neurons and orexin release show rhythmic diurnal fluctuations with a peak associated with arousal (Estabrooke et al., 2001; Fujiki et al., 2001; Yoshida et al., 2001; Saper, 2006). In addition, orexin effects on feeding and sleep-wake cycles are under circadian control and influenced by photoperiod length (Khorooshi and Klingenspor, 2005; Kantor et al., 2009; Kirsz et al., 2012). Some of the above-mentioned orexin-regulated functions dramatically change during the hibernation of small mammals including the Syrian hamster. During torpor, feeding is impossible and there is a fall in whole body metabolism and in body temperature to few degrees above ambient temperature (≤5°C). In addition, the heart beats at a much slower rate with the consequent decrease in cerebral blood flow and there is a decrease in respiratory rate (Frerichs et al., 1994; Storey, 2003; Geiser, 2004; Hampton et al., 2010). Torpor bouts are interrupted by brief periodic arousals in which these parameters are maintained for a few hours at the levels typical of euthermia before returning to torpor. In the present study, the analysis of the intensity of C-Fos and OXA immunostainings in hypothalamic orexinergic neurons of the Syrian hamster showed differential profiles in the distinct stages of hibernation. A few studies had previously explored possible changes in the orexinergic system in the different phases of hibernation. Analysis of the transcriptome in hibernating squirrels found elevated levels of mRNA orexin expression before and after hibernation, and a strong decrease in the orexinergic levels during both torpor and interbout arousals (Schwartz et al., 2013).

The general decrease found in the intensity of OXA immunostaining of orexinergic neurons during torpor is in line with the low orexin mRNA levels reported in ground squirrels during hibernation, including torpor and inter-bout arousals (Schwartz et al., 2013). In addition, this decreased OXA immunostaining intensity was accompanied by a volume reduction and fragmentation of the GA, likely related to a low capacity during torpor for processing, modification and targeting of proteins including orexins. A fragmented GA would reduce the ability to produce active orexins. Moreover, it is probably not possible for the orexins already synthesized in the rough endoplasmic reticulum at torpor to be transferred to the GA, which would explain why orexin labeling is generally observed throughout the cytoplasm and why the values for the Manders coefficient of OXA/GM-130 colocalization observed during torpor are low compared to control animals. Therefore, the fragmentation and the decrease in GA volume could involve reduction in the maturation of active orexins during torpor. It is tempting to speculate that this would affect orexinergic neurons promoting arousal, thermogenesis, feeding, and motor activity, with such neurons presumably being less active in torpor than in euthermia, and this could explain the low level of OXA immunostaining intensity observed during torpor. Fragmentation and volume reduction of the GA have also been observed in neocortical and hippocampal pyramidal neurons during torpor when they are supposedly inactive (Antón-Fernández et al., 2015).

However, it should be noted that, although the reduction of GA size has been previously associated with a decrease in neural activity (Salehi et al., 1994), we observed GA reduction and fragmentation in orexinergic neurons with different degrees of activation as judged by the expression of C-Fos (see below).

Previous studies focusing on changes in the expression of the cellular activity marker C-Fos have reported the activation of different hypothalamic nuclei during torpor and arousal phases of the hibernation of ground squirrels (Bratincsak et al., 2007). During torpor, an increase in C-Fos expression was reported in the ventrolateral division of the medial preoptic area—a thermoregulatory center—, in the suprachiasmatic nucleus that is proposed to control the rhythm of hibernation (Heller and Ruby, 2004) and also in the reticular thalamic nucleus, accompanied by a reduction of C-Fos expression in the cerebral cortex (Bratincsak et al., 2007). However, in this previous study, no attention was paid to orexinergic neurons in the LH. In the present study, we found that the activation level of orexinergic neurons in LH during euthermia, as judged by the intensity of C-Fos expression, is heterogeneous. During torpor, we also observed a general increase in C-Fos immunoreactivity in orexinergic neurons, although immunolabeling for C-Fos—and also for OXA—was variable indicating that the population of hypothalamic orexinergic neurons is heterogeneous. Previous studies have shown that orexin neurons sense more than ten neurotransmitters and hormones *via* a wide variety of receptors (Sakurai, 2007; Hara et al., 2009; Inutsuka and Yamanaka, 2013). In addition to orexin, orexinergic neurons might co-release dynorphin, glutamate and potentially nociceptin/orphaninFQ, galanin, neurotensin, pancreatin peptide islet amyloid polypeptide and GABA [for a review, see Bonnavion et al. (2016)]. Recent data suggests that hypothalamic orexinergic neurons are heterogeneous and probably segregate into distinct subpopulations operating with specific transcription factors and have differing genetic, anatomical and electrophysiological phenotypes with different responses to neurotransmitters and projection patterns (Yelin-Bekerman et al., 2015; Bonnavion et al., 2016; Sagi et al., 2021). The possible functions of orexinergic neurons with intense C-Fos immunostaining during torpor, which supposedly remain active, are unknown. However, it is unlikely that they promote sleep-wake transition, arousal or feeding, which do not operate during torpor, and we speculate that they could be related to the regulation of other physiological processes such as cardiovascular functions that, although reduced, do still have to be controlled. Orexinergic activation during torpor could also participate in the activation of the raphe nuclei which are involved in inducing and regulating the onset of the torpor cycle (Spafford and Pengelley, 1971; Canguilhem et al., 1986; Haak et al., 1991; Hitrec et al., 2019).

Orexins also participate in the coordination of BAT thermogenesis; appetite stimulation and feeding; increased physical activity; support for alertness and cognitive function necessary to sustain arousal; and exploratory and food-seeking behaviors (Sellayah and Sikder, 2013). Although it has been proposed that there might be some common mechanisms and pathway involvement between the orexin-mediated arousal from sleep and arousal from deep torpor (Sellayah and Sikder, 2013), the precise mechanisms of orexin-mediated effects on arousal from hibernation have not yet been fully explored. Previous studies showed the involvement of the hypothalamus and importance of the orexin system for arousal from hibernation. Intracerebroventricular injections of orexin and of tyrotropin-releasing hormone (TRH) interrupted hibernation in Syrian hamsters, elevating the body temperature—an effect that was inhibited by prior administration of orexin receptor antagonist, suggesting that TRH arouses hamsters through the orexin system (Tamura et al., 2011). Periodic arousals during hibernation are characterized by rewarming, with a body temperature rising to 37°C, which is maintained for 12–48 h before returning to torpor (Geiser, 2004, 2013). BAT responsiveness to adrenergic stimulation is increased and BAT thermogenesis is essential to raise the body temperature to terminate hibernation (Milner et al., 1989; Cannon and Nedergaard, 2004; Kitao and Hashimoto, 2012). In addition, OXA activity favors feeding and motor behaviors during arousal in Syrian hamsters (Sakurai et al., 1998; Avolio et al., 2012; Mele et al., 2014).

In the present study, a significant increase in the intensity of immunolabeling of OXA and C-Fos during the arousal stage of hibernation has been observed in a high proportion of orexinergic neurons in the lateral hypothalamus of the Syrian hamster. This suggests an increase in the transcriptional and translational activity of these cells that would be recovering their gene expression profile, distinct to the torpor phase. This contrasts with previous transcriptome reports showing low orexin mRNA levels during arousal in the hypothalamus of ground squirrels (Schwartz et al., 2013). Although the reason for this discrepancy is not known, the expression of RNA-binding protein RBM3, which promotes translation at mild cold body temperatures (Dresios et al., 2005), is elevated during torpor and interbout arousals in the hypothalamus (Schwartz et al., 2013). Therefore, it is possible that the increased orexin immunostaining observed during arousal in the Syrian hamster might be enhanced during the torpor-to-arousal transition when body temperature rises, despite reduced gene expression. The increased intensity of immunolabeling for OXA and C-Fos during the arousal phase (present results) is coincident with both the increase in body temperature that occurs in the exit from torpor (Geiser, 2004, 2013; Hitrec et al., 2020) and the activation of food intake, which are typical of the arousal phase and which orexins promote. Importantly, during arousal, a rapid restructuring and volume increase of orexinergic neuronal GA was observed, regaining the structural appearance and volume levels characteristic of euthermia. These changes are presumably related to increases in the synthesis and transport of functional orexins necessary for the neuronal adaptation to the torpor-to-arousal transition.

We speculate that the activation of orexinergic cells during the arousal phase of Syrian hamsters mediates the increase in thermogenesis, food intake, heart rate and blood pressure (all of which are likely to be reduced during torpor), and thereby, the orexinergic cell activation would contribute to promote behavioral arousal. Moreover, it is likely that the activity of orexinergic cells during arousal contributes to the particular sleep behavior that is characteristic of this phase of hibernation (see previous Section “Discussion”). In addition to LH orexinergic neurons, activation of other hypothalamic cell populations, as revealed by the increase in C-Fos mRNA expression, was described during arousal in the arcuate nucleus and the dorsolateral hypothalamus (areas involved in food intake), in the medial part of the preoptic area (involved in thermoregulation), and in the suprachiasmatic nucleus with a peak of C-Fos expression at the beginning of arousals (Bratincsak et al., 2007).

Finally, Djungarian hamsters do not hibernate, but when maintained at 18°C, they enter a temporary period with slightly reduced metabolism and temperature called shallow or daily torpor (Herwig et al., 2007). In Djungarian hamsters, during normothermia, general OXA mRNA expression in LH was rhythmic but, in animals which enter shallow torpor bouts, this expression was found to be lower than in normothermia and not rhythmic (Herwig et al., 2007). The lack of changes in OXA expression during daily torpor led to the suggestion thatorexin-mediated arousal pathways may not operate during natural bouts of hypothermia (Herwig et al., 2007). Other non-hibernating species, such as mice, also enter a shallow torpor state under conditions of scarce food availability and low ambient temperature with a transient metabolic suppression and slight body temperature reductions. In a recent study, it was shown that most physiological adaptations of fasting-induced shallow torpor stage in mice are preserved in OXA knock-out (KO) mice, leading to the suggestion that the physiological signature of daily torpor is mostly not orexin dependent (Lo Martire et al., 2020). However, the authors of this study reported that, as compared to wild type mice, orexin-deficient mice showed a lower brain temperature in the rewarming period, a longer duration of REM sleep before and after cooling, and longer periods of an undetermined state at the beginning of the rewarming period indicating a deficit of heat production (Lo Martire et al., 2020). Daily torpor and hibernation are different physiological processes in many regards and they might be regulated by different mechanisms and neurochemical substrates. Therefore, the differences regarding the involvement of the orexinergic system in both processes—with significant changes in orexinergic neurons during hibernation (present study), and the apparent lack of involvement of the orexinergic system throughout the phases of daily torpor—are not necessarily contrasting findings. In addition, the use of different species and methodologies might account for the differences between the present results and those reported in animals undergoing daily torpor. For example, it is possible that arousal from daily torpor is too fast to be reflected in changes in OXA mRNA levels. Even so, additional studies are needed in the future to clarify and fully characterize the complex biological processes involved in hibernation.

## Data availability statement

The raw data supporting the conclusions of this article will be made available by the authors, without undue reservation.

## Ethics statement

This animal study was reviewed and approved by Animal Experiment Ethics Committee (PROEX 292/15).

## Author contributions

GL-E generated the experimental groups of animals. PC and JP performed experiments and analysis. JL and AM designed the study, made figures, interpreted the results, and wrote the manuscript. All authors revised the manuscript, contributed to the article, and approved the submitted version.

## Footnotes

1
https://imagej.nih.gov/ij/
